# A Bacterial Toxin with Analgesic Properties: Hyperpolarization of DRG Neurons by Mycolactone

**DOI:** 10.3390/toxins9070227

**Published:** 2017-07-18

**Authors:** Ok-Ryul Song, Han-Byul Kim, Samuel Jouny, Isabelle Ricard, Alexandre Vandeputte, Nathalie Deboosere, Estelle Marion, Christophe J. Queval, Pierre Lesport, Emmanuel Bourinet, Daniel Henrion, Seog Bae Oh, Guillaume Lebon, Guillaume Sandoz, Edouard Yeramian, Laurent Marsollier, Priscille Brodin

**Affiliations:** 1 Univ. Lille, CNRS, Inserm, CHU Lille, Institut Pasteur de Lille, U1019-UMR8204-CIIL-Center for Infection and Immunity of Lille, F-59000 Lille, France; ok-ryul.song@ibl.cnrs.fr (O.-R.S.); samuel.jouny@inserm.fr (S.J.); isabelle.ricard@ibl.cnrs.fr (I.R.); alexandre.vandeputte@ibl.cnrs.fr (A.V.); nathalie.deboosere@pasteur-lille.fr (N.D.); cj.queval@gmail.com (C.J.Q.); 2 Pain Cognitive Function Research Center, Department of Brain and Cognitive Sciences College of Natural Sciences, Dental Research Institute and Department of Neurobiology and Physiology, School of Dentistry, Seoul National University, Seoul 110-799, Korea; u4588719@snu.ac.kr (H.-B.K.); odolbae@snu.ac.kr (S.-B.O.); 3 CRCINA, INSERM, Université de Nantes, Université d'Angers, 4 rue Larrey, F-49933 Angers, France; estelle.marion@inserm.fr; 4 Equipe ATIP AVENIR, CRCINA, INSERM, Université de Nantes, Université d'Angers, 4 rue Larrey, F-49933 Angers, France; 5 Laboratories of Excellence, Ion Channel Science and Therapeutics, Institut de Génomique Fonctionnelle, 141 rue de la Cardonille, 34094 Montpellier, France; pierre.Lesport@igf.cnrs.fr (P.L.); emmanuel.Bourinet@igf.cnrs.fr (E.B.); 6 CNRS UMR5203, 141 rue de la Cardonille, 34094 Montpellier, France; 7 INSERM U1191, Univ. Montpellier, 141 rue de la Cardonille, 34094 Montpellier, France; 8 UMR CNRS 6214-INSERM 1083, Laboratoire de Biologie Neurovasculaire et Mitochondriale Intégrée, UFR Sciences Médicales, Université d’Angers, Rue Haute de Reculée, 49045 Angers, France; daniel.henrion@univ-angers.fr; 9 INSERM U1191, CNRS UMR 5203, Institut de Génomique Fonctionnelle, 141 rue de la Cardonille, F-34094 Montpellier, France; guillaume.Lebon@igf.cnrs.fr; 10 Laboratories of Excellence, Ion Channel Science and Therapeutics, UMR 7277, Institute of Biology Valrose (iBV), Université Nice Sophia Antipolis, F-06100 Nice, France; guillaume.sandoz@unice.fr; 11 Unité de Microbiologie Structurale, CNRS UMR3528 Institut Pasteur, 75015 Paris, France

**Keywords:** Buruli ulcer, mycolactone, AT_2_ receptors, DRG neurons, membrane potential, image-based assay, high-content screening

## Abstract

Mycolactone, a polyketide molecule produced by *Mycobacterium ulcerans*, is the etiological agent of Buruli ulcer. This lipid toxin is endowed with pleiotropic effects, presents cytotoxic effects at high doses, and notably plays a pivotal role in host response upon colonization by the bacillus. Most remarkably, mycolactone displays intriguing analgesic capabilities: the toxin suppresses or alleviates the pain of the skin lesions it inflicts. We demonstrated that the analgesic capability of mycolactone was not attributable to nerve damage, but instead resulted from the triggering of a cellular pathway targeting AT_2_ receptors (angiotensin II type 2 receptors; AT_2_R), and leading to potassium-dependent hyperpolarization. This demonstration paves the way to new nature-inspired analgesic protocols. In this direction, we assess here the hyperpolarizing properties of mycolactone on nociceptive neurons. We developed a dedicated medium-throughput assay based on membrane potential changes, and visualized by confocal microscopy of bis-oxonol-loaded Dorsal Root Ganglion (DRG) neurons. We demonstrate that mycolactone at non-cytotoxic doses triggers the hyperpolarization of DRG neurons through AT_2_R, with this action being not affected by known ligands of AT_2_R. This result points towards novel AT_2_R-dependent signaling pathways in DRG neurons underlying the analgesic effect of mycolactone, with the perspective for the development of new types of nature-inspired analgesics.

## 1. Introduction

*Mycobacterium ulcerans* is the causative agent of Buruli ulcer (BU), a severe infective skin disease, which now represents the third most common mycobacterial disease in the world, after tuberculosis and leprosy [1,2]. This skin disease that has re-emerged in the last two decades affects mainly children, and provokes lesions that can often lead to permanent disabilities [3]. Epidemiological studies have shown that aquatic environments represent the main reservoir of *M. ulcerans* [4,5]. *M. ulcerans* is able to colonize various ecological niches, from aquatic environments to humans. Indeed, in aquatic environments, numerous aquatic vertebrates and macroinvertebrates harbor the bacilli. The exact ecology and mode(s) of transmission of *M. ulcerans* to humans need still to be fully specified.

In Buruli ulcer disease, skin lesions are caused by a toxin, called mycolactone, the main virulence factor of *M. ulcerans* [6]. As such, the secretion of this toxin represents a unique and distinctive feature characterizing *M. ulcerans*, playing a central role in its eco-epidemiology and pathogenesis [7,8]. After penetration in the skin, the lifecycle of *M. ulcerans* includes an intracellular phase, allowing the bacillus to evade host immune system recognition. Upon secretion of mycolactone, *M. ulcerans* enters then an extracellular stage, with a massive destruction of host tissue, caused by a local increase in the concentration of the toxin.

In fact, beyond its cytotoxic properties, the pleiotropic effects exerted by the toxin are observed in these two main lifecycle stages, with mycolactone modulating the immune system, influencing the production of cytokines, and acting on the peripheral nervous system, rendering the skin lesions painless [8].

The singularity of the analgesia exerted by mycolactone resides in the underlying signaling pathway, which we unraveled recently [9]. More specifically, we demonstrated that mycolactone induces analgesia, without nerve damage, by targeting angiotensin pathways, leading to the potassium-dependent hyperpolarization of neurons upon binding to angiotensin II type 2 receptors (AT_2_R). The absence of nerve damage was formally demonstrated in animal models by inoculation with mycolactone, with animals recovering their sensibility 48 h after injection. Altogether, our results and observations strongly suggest that the cytotoxic and analgesic effects of mycolactone are mediated by distinct pathways.

In our previous study, we demonstrated that mycolactone, targeting angiotensin pathways, is capable of triggering potassium-dependent hyperpolarization in cells of different types, including macrophages, PC12 pheochromocytoma cells, and hippocampal neurons [9]. However, this study did not include nociceptive-specific cells. It is then mandatory, in the way towards the development of mycolactone-inspired analgesics, to extend the studies to this type of sensory cell. In this context, we assess here the capability of mycolactone to hyperpolarize Dorsal Root Ganglion neurons (DRG neurons). Importantly, in this background, it appears that AT_2_R is indeed expressed in DRG neurons, as typically isolated, for in vitro studies, from the spinal cord of rats [10], of mice [11], or from injured nerve specimens from adult humans [12].

In order to conduct our study on the action of mycolactone on DRG neurons, we developed a dedicated medium-throughput assay, which allows for the monitoring of the changes of membrane potentials in a large number of DRG neurons. More specifically, this streamlined assay relies on the automated image analysis of membrane potential changes, visualized by confocal microscopy of bis-oxonol-loaded DRG neurons. As this development, beyond our specific objective, can be valuable for its own sake for various medium-throughput assays of neuroactive compounds on DRG neurons, we first detail the methodology on general grounds, pointing out various advantages in this context as compared to the patch-clamp approach, the gold standard technique for the analysis of the electrical properties of neurons. In this respect, above all, using only a very small number of mice (typically 4–6), our medium-throughput miniaturized assay allows for studies to easily analyze more than 100 different conditions, with about 400 DRG neurons for any given condition. By comparison, with electrophysiology, studies would be typically limited to significantly lower numbers of different conditions and cells. Using our streamlined assay, we address the question of the action of mycolactone on DRG neurons. We demonstrate that, at non-cytotoxic doses, mycolactone triggers hyperpolarization of DRG neurons through AT_2_R.

Finally, our study can be further envisioned in the more general context of the search for nature-inspired analgesic solutions. Indeed, as largely emphasized recently, such approaches appear to represent, in the search for new analgesics, promising alternatives to the traditional pharmacological strategies, whose limitations have become increasingly apparent (as well summarized by the title of an editorial review in the Lancet journal: “Results in Analgesia—Darwin 1, Pharma 0”) [13]. In this context, we discuss the perspectives opened by our study for the development of new analgesics inspired by the natural solution for analgesia implemented in Buruli ulcer.

## 2. Results

### 2.1. Development of a Medium-Throughput Visual Assay of DRG Neurons

**Figure 1:**
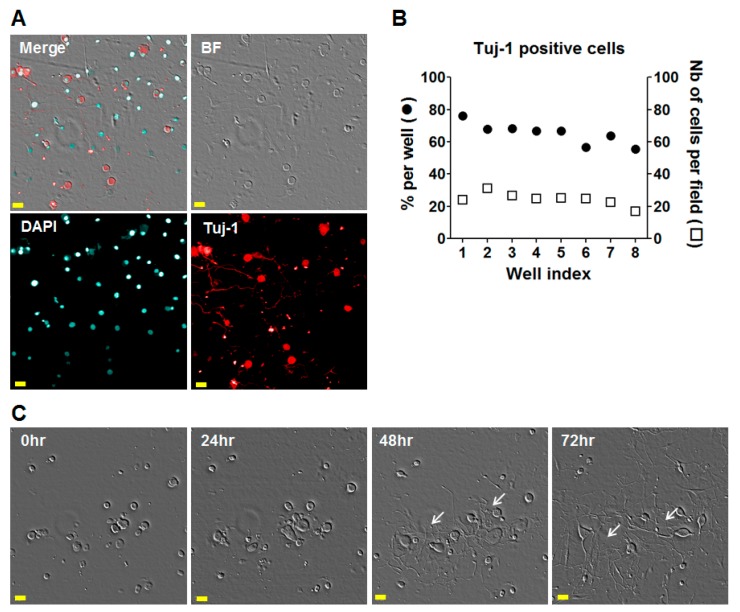
Morphology of the Dorsal Root Ganglion (DRG) neurons 3 days after harvesting in 384-well microplates. (**A**) Bright-Field (BF) and confocal images. DRG neurons were labelled for Tuj-1 monoclonal antibody, allowing the detection of neuron-specific beta-tubulin III. DAPI-staining was used for nucleus labelling. Scale bar = 20 µm; (**B**) Percentage of Tuj-1 positive cells per well and number of Tuj-1 positive cells per field. The percentage of Tuj-1 positive cells was determined for 200 cells in average; (**C**) BF images of DRG neurons at 0, 24, 48, and 72 h after plating in microplate; Scale bar = 20 µm; White arrow, neurite growth.

To assess the ability of mycolactone to induce the hyperpolarization of nociceptive DRG neurons, we developed a dedicated, streamlined, automated assay. More precisely, we established and optimized a DiSBAC_2_(3) image-based automated confocal microscopy assay, relying on the monitoring of membrane potentials. The development focused mainly on the miniaturization of the automated assay, enhancing the quality of data, and reducing the number of mice (see Materials and Methods) needed per experiment. To achieve this challenge, we optimized the conditions of miniaturization for dissociated DRG neuron cultures in 384-well micro-titer plates. More specifically, for the optimizations, several parameters were monitored, including those relevant to the methods of dissociation, the density of DRG neurons per well, the enrichment in DRG population, and the presence of neurites. Indeed, the cell density parameter is essential for the image acquisition protocol with our automated microscope as well as for ensuring proper neuronal connectivity. Our optimization process thus led us to set the number of seeded cells per well to 4000, which consisted in a mixed population of DRG neurons and non-neuronal cells. After three days of selection using Ara-c and FdU to impede the survival of non-neuronal cells, images were acquired on the confocal microscope with a 20× magnification, leading to the detection of about 20 to 30 small- and medium-sized DRG neurons per image. In order to determine the exact number of DRG neurons in a microplate at this time point, the cells were stained for β-III tubulin using Tuj-1 antibody, and we found that, on average, 65% of detected cells were Tuj-1 positive (Figure 1A,B). Thus, this system typically allows for the analysis of more than 100 different conditions with a very small number of mice (typically 4–6). In parallel, the health of cultured DRG neurons was assessed by monitoring neurite development. As shown in Figure 1C, the neurite growth of the DRG neurons was assessed by bright-field time-course imaging, after 48 h and 72 h of culture, with neural connectivity estimated based on cell density.

### 2.2. Monitoring Membrane Potentials of DRG Neurons with DiSBAC_2_(3)

We adapted the procedures relying on the use of DiSBAC_2_(3) as a fluorescent indicator to monitor the membrane potentials of DRG neurons with our miniaturized visual automated assay.

In our previous studies concerning macrophages and hippocampal neurons [9], we have suggested the use of DiSBAC_2_(3) at 150 µM concentration. However, in the present case, image analysis becomes difficult and cumbersome at such a concentration, as the DRG neurons are saturated by DiSBAC_2_(3). In addition, in such conditions the non-neuronal cells were also stained by DiSBAC_2_(3) (data not shown).

**Figure 2:**
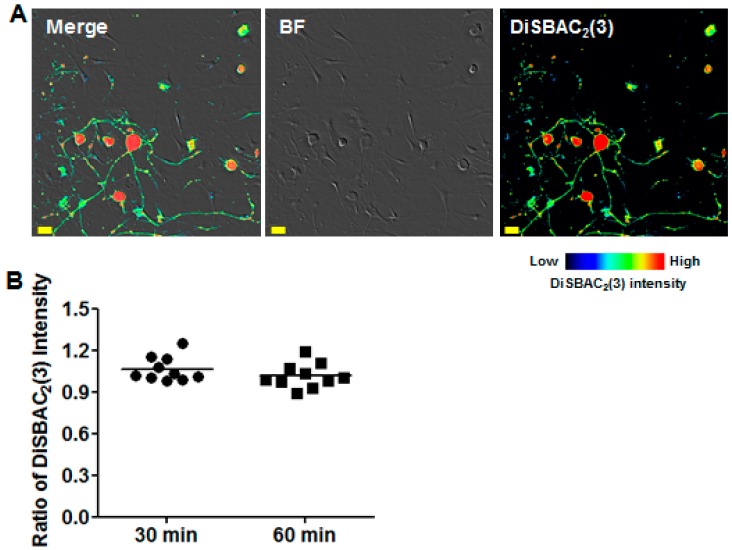
Stability of DiSBAC_2_(3) intensity in DRG neurons. (**A**) Bright-Field (BF) and confocal image of DRG neurons loaded with 25 µM DiSBAC_2_(3); Scale bar = 20 µm; (**B**) Quantification of DiSBAC_2_(3) intensities presented as ratios: the signal intensities were calculated by normalizing the DiSBAC_2_(3) intensity at 30 min and 60 min to that before addition; each point corresponds to the mean value over 10 wells (corresponding to at least 1200 cells).

To alleviate these difficulties, we tested different concentrations of DiSBAC_2_(3). The tests allowed us to determine the appropriate concentration of DiSBAC_2_(3) (25 µM) to ensure a proper image analysis of DRG neurons (Figure 2A). Indeed, at such concentration, the fluorescence intensity level of non-neuronal cells appears to be low (because of the differences in the native resting membrane potentials for the various cell types), making possible its elimination as a background level using image analysis software (Figure S1). In addition, it was possible to ascertain the stability of DiSBAC_2_(3) staining intensity over a one hour period (Figure 2B). This stability renders our system suitable for the study of compounds with long-lasting effects on DRG membrane potentials, with the reproducible assays displaying high sensitivity to small variations of membrane potentials.

**Figure 3:**
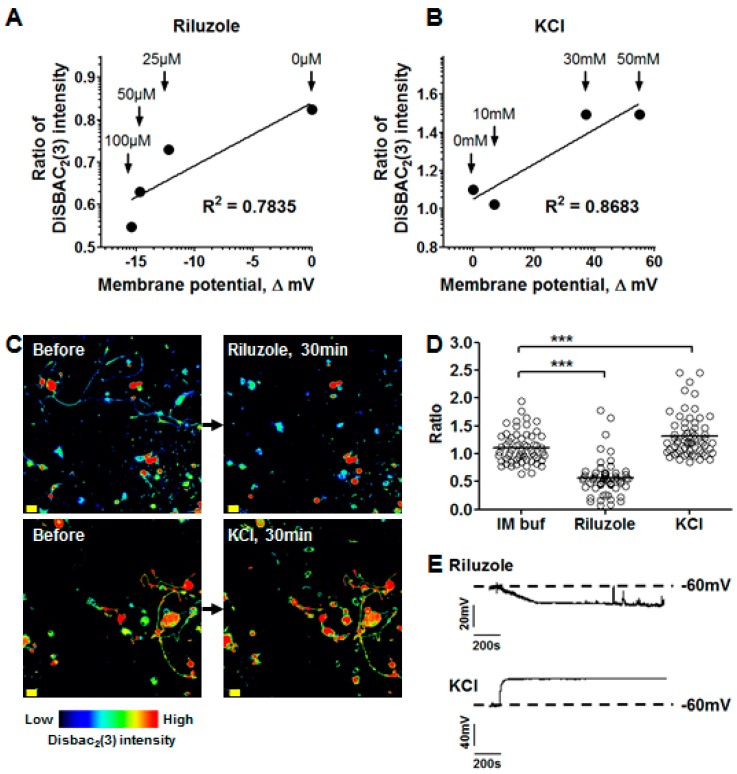
Change in membrane potential in DRG neurons. (**A**,**B**) Membrane potential measured by patch-clamp experiments (X-axis, *n* = 5 cells) and DiSBAC_2_(3) assays (Y-axis, *n* >180 cells) for Riluzole (**A**) at concentrations of 0, 25, 50, and 100 μM and for KCl (**B**) at concentrations of 0, 10, 30, and 50 mM. The ΔmV and the ratio of DiSBAC_2_(3) were calculated by normalizing the fluorescence intensity at 20 min to that at 0 min; (**C**) Images of DRG neurons before and after addition of Riluzole (100 µM) or KCl (50 mM); Scale bar = 20 µm; (**D**) Change in membrane potential after addition of Riluzole (100 µM) or KCl (50 mM). The ratio was calculated by normalizing the intensity at 20 min to that at 0 min; *** *p*-value < 0.0001; (**E**) Representative chart recording traces of the membrane potential of DRG neurons treated with Riluzole (100 µM) or KCl (50 mM).

More specifically, we showed that our miniaturized assay allows for the visualization of membrane potential changes by treating DRG neuron cultures with KCl (depolarizing agent) [14] or with Riluzole (hyperpolarizing agent) [15,16]. The dose-response curves of the compounds with Riluzole and KCl in DiSBAC_2_(3) assays on DRG neurons (Figure 3A,B) showed that (1) Riluzole triggers a decrease in cell fluorescence below 25 µM, indicative of cell hyperpolarization, and (2) KCl triggers an increase in cell fluorescence above 30 mM, indicative of cell depolarization. Maximal effects were observed at 100 µM Riluzole and at 50 mM KCl (Figure 3C,D). It is worth noting that a major strength of our assay, with the imaging time of 0.07 ms per field, resides in the possibility to compare diverse conditions on the same plate. For further validation of our image analysis process, the assays were paralleled by patch-clamp experiments (Figure 3E). The results showed that slow acting drugs such as Riluzole induced the gradual hyperpolarization of membranes for approximately 8 min after the compound’s addition, with the effect plateauing after that time. On the other hand, the high concentration of KCl induced the rapid depolarization of membranes, with the effect plateauing 1 min after the KCl’s application. In Figure 3, the fluorescence data are plotted on the corresponding values for membrane potentials, as described by a previous study [17]. Briefly, the maximal effect of Riluzole is associated with a decrease of about 44% in cell fluorescence, corresponding to a variation of 15 mV as recorded by patch-clamp. On the other hand, KCl provokes an increase of about 32% in cell fluorescence, corresponding to a variation of 55 mV as recorded by patch-clamp (Figure 3D,E). Our data are in accordance with other studies using DiSBAC_2_(3) to visualize membrane potential changes.

### 2.3. Mycolactone Induces Hyperpolarization of DRG Neurons at Non-Cytotoxic Doses

In our previous study concerning macrophages, PC12 cells, and hippocampal neurons, we showed that mycolactone, at non-cytotoxic doses, induces membrane hyperpolarization [9]. More specifically, mycolactone induced cell hyperpolarization by targeting angiotensin pathways, and binding to AT_2_R. Based on these results, we made the hypothesis that hyperpolarization induced by mycolactone upon binding to AT_2_R, could cause local analgesia, and we confirmed this hypothesis in an animal model. With the methodological development above, we can extend the study of the effect of mycolactone to DRG neurons involved in pain signals.

**Figure 4:**
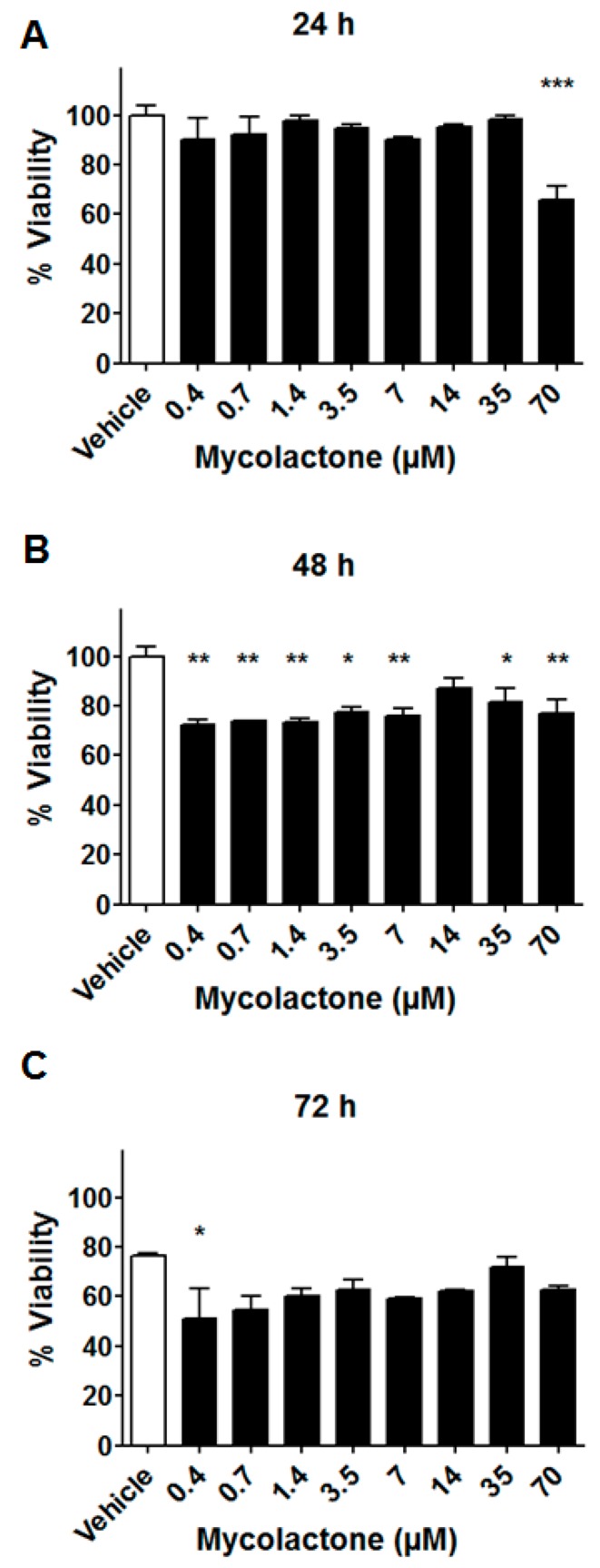
Time dependence of the effect of mycolactone on the viability of DRG neurons. Viability of DRG neurons after different incubation times with mycolactone: (**A**) 24 h, (**B**) 48 h, and (**C**) 72 h. Each bar corresponds to the mean value of two wells (that have been harvested with 600 cells). *p*-values were calculated by ANOVA using Dunnett’s Test for multiple comparisons to the vehicle control, *** *p*-value < 0.001; ** *p*-value < 0.01; * *p*-value < 0.05.

For DRG neurons, we first evaluated the cytotoxic effect of mycolactone, for concentrations ranging between 0.4 µM and 70 µM, over a three day period using the Toxilight bioassay kit. For the tested concentration range, no significant cytotoxic effect was observed at 24 h post-addition of mycolactone (Figure 4A). At 48 h, 80% of the DRG neurons were still viable up to the maximal tested concentration of 70 µM (Figure 4B). At 72 h, the vehicle (DMSO 0.2%) started to exhibit toxicity, with about 80% of viable cells, whereas about 60% of DRG neurons remained viable for the mycolactone samples at all of the tested concentrations (Figure 4C). DRG neurons cultured in 384-well plate did not survive to 96-hour incubation after 3-day seeding. As a matter of fact, under such conditions, most of the DRG nuerons had died in the vehicle control (less than 20% viability), making it impossible to assess the cytotoxic effects of mycolactone at this time point.

Altogether, our data showed that DRG neurons are far less sensitive to the cytotoxic effects of mycolactone compared to other cell types (Table 1). Similar conclusions were reached for hippocampal neurons [9]. On the other hand, with the exception of T lymphocytes, HEK293T, and Huh7, mycolactone appears to kill many different types of cells at such concentration (Table 1).

**Figure 5:**
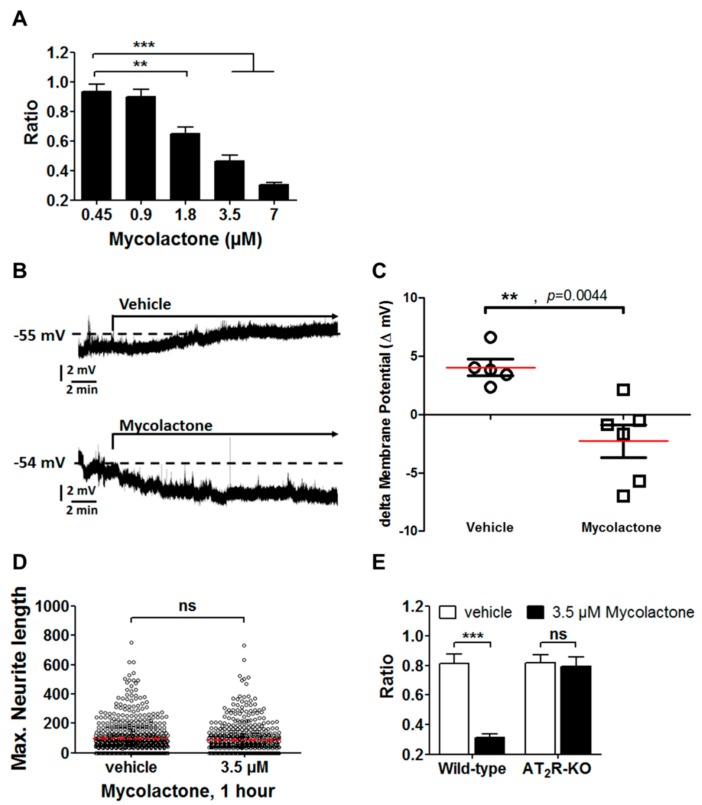
Characterization of the effects of mycolactone on DRG neurons. (**A**) Hyperpolarization of DRG neurons upon addition of mycolactone at different concentrations. The ratio corresponds to the intensity of the DiSBAC_2_(3) dye before addition and after one hour from the addition of mycolactone. A ratio lower than 0.6 corresponds to hyperpolarization; (**B**,**C**) Membrane potential recording by patch-clamp. Representative chart recordings of the membrane potential of DRG neurons challenged either by 0.2% DMSO (vehicle) or mycolactone (3.5 µM) (**B**). Pooled data illustrating hyperpolarization triggered by continuous application of mycolactone for 20 min. Red line: mean (**C**); (**D**) Maximum neurite length in Tuj-1 positive neurons incubated with (*n* = 499) or without (*n* = 583) mycolactone; Mann–Whitney test; ns, not significant; Red dot line, mean; (**E**) Quantification of the DiSBAC_2_(3) ratio for DRG neurons from wild-type and AT_2_R deficient (AT_2_R-knock-out (KO)) mice after loading with 3.5 µM mycolactone for 1 h; *** *p*-value < 0.0001; ** *p*-value < 0.001; ns, not significant.

Next, we showed in Figure 5A that mycolactone was able to hyperpolarize DRG neurons at concentrations above 1 µM. The level of hyperpolarization induced by mycolactone is dose-dependent. No effect could be observed at submicromolar concentrations. The maximal effect was recorded at 30 min post-addition of mycolactone and remained maximal during one hour. In all subsequent experiments, the dose of mycolactone was set at 3.5 µM, checked to be non-cytotoxic even after 24 h of exposure (Figure 4A), with the hyperpolarization effect further validated by patch-clamp experiments (Figure 5B,C). In addition, monitoring the neurite growth showed that exposure to mycolactone at a concentration of 3.5 µM for 60 min did not affect neurite lengths (Figure 5D). Furthermore, expectedly, mycolactone did not induce hyperpolarization in DRG neurons isolated from AT_2_R-knock-out mice (Figure 5E and Figure S2). Altogether, our data show that mycolactone induces hyperpolarization in DRG neurons targeting specifically angiotensin II type 2 (AT_2_) receptors.

We then wanted to further characterize in DRG neurons the specific interaction between mycolactone and AT_2_R, leading to cell hyperpolarization. To pinpoint the specificities of mycolactone/AT_2_R interactions, we tested in our assay known ligands of AT_2_R: (i) angiotensin II; (ii) EMA 401, known to alleviate neuropathic pain through AT_2_R; and (iii) C21 endowed with anti-inflammatory capabilities, targeting AT_2_R (Table 2) [12,26,27].

**Figure 6:**
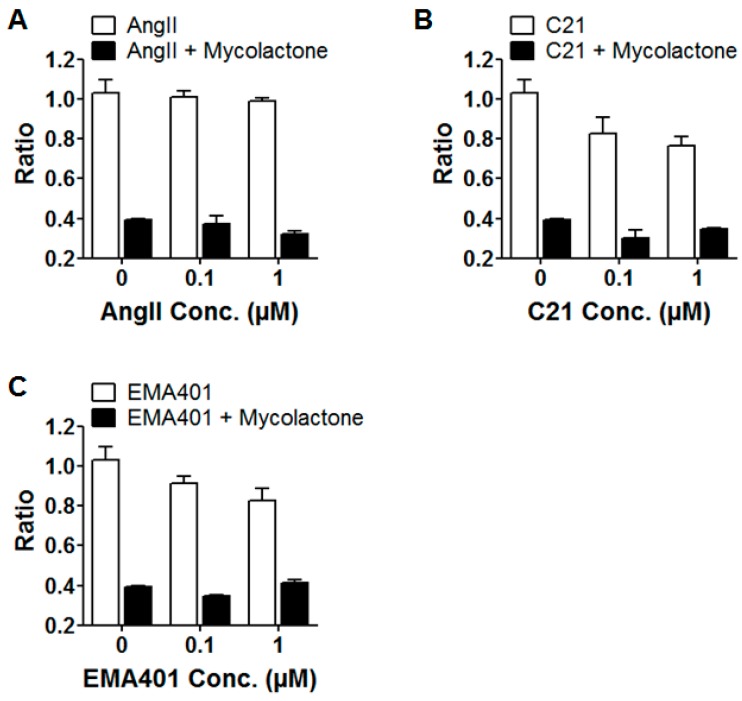
Effect of high affinity ligands to AT_2_R on DRG neurons. (**A**–**C**) DiSBAC_2_(3) fluorescence of DRG neurons calculated as the ratio before and after treatment with ligands for 30 min (white bar). After 30 min incubation with the compound, mycolactone at 3.5 µM was added for 30 min (black bar); (**A**) Angiotensin II, (**B**) C21, or (**C**) EMA401; minimum two wells per condition were analyzed (including at least 200 cells).

As shown in Figure 6, all of these compounds are not able to trigger the hyperpolarization of DRG neurons, as a difference to mycolactone. Moreover, the co-incubation of EMA 401 and C21 with mycolactone did not affect the hyperpolarization mediated by mycolactone. Altogether, these results further demonstrate that the mycolactone-mediated signaling that triggers hyperpolarization is not inhibited by these AT_2_R ligands.

## 3. Discussion

*Mycobacterium ulcerans* is the causative agent of Buruli ulcer, a neglected and emerging infectious disease [2,19]. This infection often provokes several massive cutaneous ulcers [1]. A singularity of the lesions resides in the lack of pain, with, as a consequence, patients not seeking appropriate medical care. In Buruli ulcer, the lesions are caused by mycolactone, the main known virulence factor of *M. ulcerans*. Mycolactone has been associated with various effects (such as the induction of cell death or immunomodulatory effects) [8]. Mycolactone was shown to act on different types of cellular receptors and signaling pathways [20]. It has first been shown by Demangel and colleagues that mycolactone induces skin lesions by hijacking the Wiskott–Aldrich syndrome protein [28]. Concomitantly, Simmons and colleagues reported that mycolactone-induced cytotoxicity was caused by the inhibition of Sec61-dependent translocation, leading to profound changes in cellular signaling [29]. These findings were further substantiated by a report by Baron et al. demonstrating that Sec61 is the host receptor for the mycolactone-mediated immunomodulation, leading to the current understanding that Sec61 is the major target underlying the cytotoxicity of mycolactone [30]. Furthermore, mycolactone was shown to promote Bim-dependent apoptosis via the mTor pathway [20]. It is thus very likely that, as a multi-target weapon, mycolactone triggers several signaling pathways leading to massive skin destruction but also to analgesic effects.

In this background, we have recently demonstrated that mycolactone can induce local analgesia, and the expression of AT_2_R is required for the painlessness of lesions [9]. We have thus attributed this effect to the capability of mycolactone to trigger a sustained hyperpolarization of neurons. Moreover, we have identified the detailed cellular pathway underlying this effect, showing that hyperpolarization is triggered upon the binding of mycolactone to AT_2_ receptors, leading to the activation of TRAAK channels [9]. Overall, our results suggested that the implementation of analgesia in Buruli ulcer could inspire the development of new efficient analgesics, based on the properties of mycolactone acting on the AT_2_R/TRAAK system. In such a context, it was then mandatory to extend our studies to DRG neurons, the sensory neurons involved in pain.

Here, we characterized the effect of mycolactone on DRG neurons, acting on the AT_2_ receptor. We thus adapted the fluorescence based that we used to identify the signaling pathway involved in mycolactone mediated hyperpolarization to DRG neurons [9].

**Figure 7:**
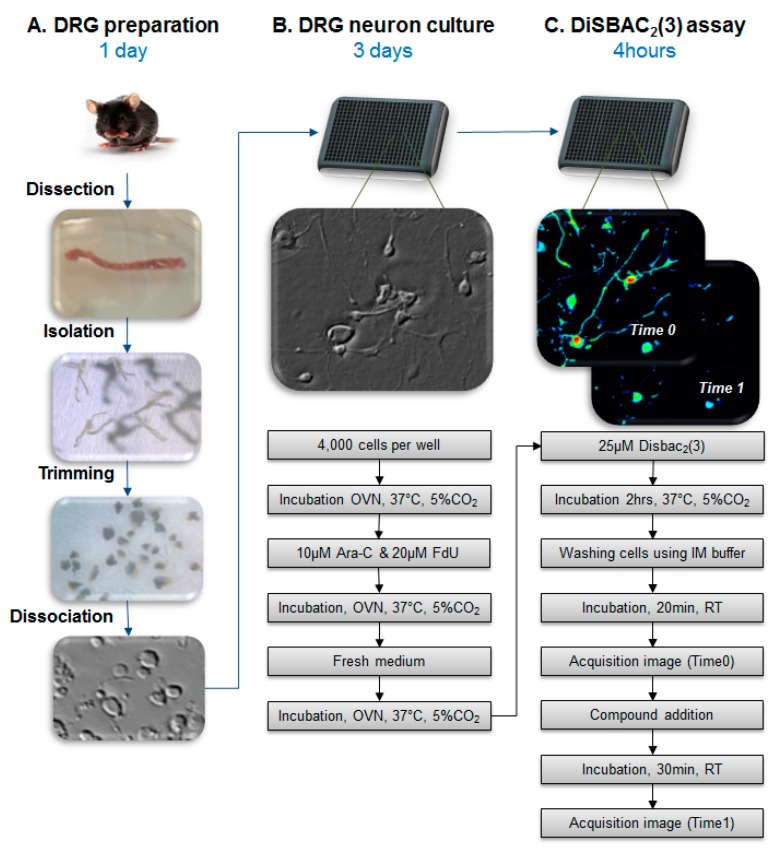
Schematic overview of dorsal root ganglion (DRG) culture and DiSBAC_2_(3) assay in a micro-titer plate. (**A**) DRG preparation; (**B**) DRG neuron plating and culture during three days; (**C**) DiSBAC_2_(3) assay in the micro-titer plate.

On methodological grounds, we developed an efficient medium-throughput high-content imaging assay (Figure 7), which can represent a powerful alternative to electrophysiology. As such, this development can be valuable for its own sake in studies aiming to understand the physiology of DRG neurons and/or to identify active compounds modulating the membrane potentials. Our work allowed for pinpointing the major advantages gained with the development of the imaging assay as compared to the patch-clamp technique. These advantages can be conveniently summarized following three main axes:

(1) Accessible scope: from low-throughput to medium-throughput

A major strength of the DiSBAC_2_(3)-based high-content imaging assay resides in the quantitative jump in the scope of analysis made possible, concerning the number of cells and the number of different conditions. Thus, with the patch-clamp technique the scope of analysis is typically limited to 15 cells per experience (and per day). Such limitation makes it difficult to implement a large number of control conditions. By comparison, the imaging assay permits a medium-throughput scope, with the possibility to implement typically 100 different conditions, with 400 cells per condition. It becomes then possible to envision the routine screening of small-size libraries, and performing dose-response tests, with the possibility of several replicates for each assay.

(2) Accessible cell sizes

In the patch-clamp technique the size of cells accessible to the experiments is limited by the size of electrodes, thus impeding for example the analysis of neurites. By comparison, no such limit exists with the imaging assay, and notably it becomes perfectly feasible to analyze the membrane potentials of neurites.

(3) Miniaturization and homogenization of experimental conditions

A notable advantage of the imaging assay is that it allows the miniaturization of the experimental conditions in terms of the volumes of the test solutions. Thus, the volumes required are typically in the range of 50 µL, as compared to the bulk test solutions required for patch-clamp which are typically in the range of 500 µL per reservoir for one cell. Such an advantage can be critical, notably when the tested compounds are available in small quantities. On the other hand, the experimental settings in the imaging assay allow for the obtaining of a high degree of homogeneity in the recording conditions, with typically 400 cells analyzed in the same conditions. By comparison, such homogeneity is not achieved with the patch-clamp technique, with the possibility for each cell to be analyzed in different environmental conditions (such as for temperature).

In addition, beyond the various technical considerations above, it is worth highlighting the easiness of use of the imaging assay, as compared to the skilled experience required for the patch-clamp technique. Nevertheless, of course for certain types of specific analyses, patch-clamp is the technique of choice, for example when high sensitivity (pA resolution) is required, or for noise measurements of currents passing through low-conductance (pS) channels.

Concerning the effect of mycolactone on DRG neurons, we demonstrated using our method that non-cytotoxic doses provoke hyperpolarization of the neurons, targeting AT_2_R. Interestingly, our results showed that the DRG neurons were less prone than other cell types to the cytotoxic effects of mycolactone. (Table 1). We show that AT_2_R is not involved in mycolactone-mediated cell death, as the viability of DRG neurons from AT_2_R-deficient mice is similar to that of wild-type mice upon mycolactone addition.

It is worth mentioning that our results are in contrast with those reported by Anand et al. [26], notably concerning the cytotoxicity of mycolactone in DRG neurons. More precisely, in this study, the cytotoxicity result appears as part of a demonstration essentially aiming to invalidate the analgesic effect of mycolactone targeting specific pathways, favoring the model of nerve destruction similar to the leprae case.

In this respect, Anand et al. report that mycolactone induces a loss of neurites in DRG neurons after incubation at 0.1 µM for 24 h, whereas we do not observe such an effect in our settings up to 35 µM (data not shown). Indeed, such effect on neurite outgrowth is typically used as a correlate of neuronal cytotoxicity. In addition, it was also reported in this study that, at 48 h, mycolactone-treated cultures displayed reduced Gap43 and β-tubulin expressions, as well as mitochondrial clumping, which further correspond to indications of cytotoxicity.

At present, it is not possible to account straightforwardly for such discrepancies between the two studies, and only further experiments, notably by other teams, could help clarify the situation.

It is nevertheless relevant to notice that the study by Anand and colleagues concerned human and rat DRG neurons. Even though not very plausible, one possible explanation for the discrepancy between the two studies may come from different sensitivity to the cytotoxicity of DRG neurons, following species. Beyond this argument concerning cytotoxicity, it is interesting to notice that in their demonstration, Anand et al. invoke the absence of the effect of Angiotensin II or the AT_2_R antagonist EMA401 in their settings to hint that mycolactone could in effect not target the AT_2_R receptors. As a matter of fact, our study fully accounts for this observation, as we demonstrated that, as a difference to other known ligands of AT_2_R (agonists or antagonists, including Angiotensin II, C21, and EMA 401) [12,26,27], only mycolactone appears able to trigger hyperpolarization of DRG neurons. Moreover, we demonstrated that EMA 401 or C21 do not inhibit hyperpolarization triggered by mycolactone. This observation pinpoints the specificity of mycolactone/AT_2_R interactions. On the other hand, interestingly, the cellular mechanisms reported to account for mycolactone-induced cell death involve hijacking different signaling pathways, such as the mTOR pathway or the pathway involving Sec61-dependent protein translocation into the ER [20,30]. This last signaling was shown to occur in cells which are susceptible to very low doses of mycolactone at 48 h in in vitro conditions [20,21]. Such results then raise the possibility that mycolactone in DRG neurons could interfere with mTOR and Sec61 pathways.

Concerning cell types, we observed the hyperpolarizing effect of mycolactone in various cell types, including neuronal cells (macrophages, pheochromocytoma cells (PC12), and hippocampal neurons). In PC12, we found a hyperpolarization of −10 mV on average [9], while in DRG the hyperpolarization was of −7 mV. However, using the fluorescence-based assay, essentially identical ratio values were obtained for these two types of cells. These data suggest that there is no difference between the two types of cells concerning sizes of responses. In terms of doses of mycolactone, the PC12 cells appeared to be more sensitive than the DRG neurons. The explanation behind this difference requests further investigation.

Finally, in the perspective of the implementation of analgesia protocols, two important issues relative to practical conditions (concentrations) as well as fundamental mechanisms (AT_2_R signaling) will need to be considered in detail.

Concerning the concentrations issue, it was reported by Sarfo and colleagues that the concentrations of mycolactone detected in lesions of BU patients are in the mid nM range [31,32]. Such concentration is about one hundred fold lower than what is required to trigger hyperpolarization in DRG neurons in vitro. However, the apparent high dose of mycolactone required to induce hyperpolarization may be overestimated due to experimental biases such as the strong affinity of mycolactone to plastic wares (including tips and microplate), thus limiting the effective amount of mycolactone available for the cell assay. There is also likely an issue with the stability of mycolactone in cell culture media, as well as upon light exposure [33]. On the other hand, in tissues, upon infection, mycolactone is released by the bacilli within vesicles [34], with the possibility for concentration gradients occurring in the lesions. Noticeably, so far, mycolactone quantification relies on the total lipid extraction of skin biopsies or on whole blood quantification [31,32], and, accordingly, the local concentrations of mycolactone at the DRG neurons’ contacts may be underestimated.

Concerning the fundamental mechanisms, there is at present a lack of knowledge on the signaling of AT_2_R, and no functional assays are available to monitor AT_2_R activation in HEK cell models, for instance. The specificities of the receptor (such as the absence of G-coupling or internalization) have been recently brought under new light with the elucidation of AT_2_R’s crystal structure [35]. In this context, it does not appear possible to rank the activity of AT_2_R ligands based on their binding affinity, and as stated in a recent review, the molecular mechanisms by which AT_2_R blockers mediate analgesia remain to be fully elucidated [36]. The perspectives opened by our study for the development of new analgesics will then be embedded in the endeavors to characterize in detail the pharmacology and specificities of AT_2_R receptors and pathways.

## 4. Materials and Methods

### 4.1. Materials

DMEM medium, Neurobasal A medium, F-12 medium, Fetal Bovine Serum (FBS), HBSS, D-PBS, Penicillin/Streptomycin, B-27, Collagenase II, Dispase II, Trypsin, Alexa Fluor^®^ 647 conjugated anti-rabbit antibody, and DiSBAC_2_(3) were purchased from Life Technologies (Carlsbad, CA, USA). DNAse 1, 30% BSA, Cytosine β-d-arabinofuranoside hydrochloride (ARA-c), 5-Fluoro-2′-deoxyuridine 5′-monophosphate sodium salt (FdU) solution (FdU), poly-d-Lysine, DAPI, and 10% neutral buffered formalin solution were purchased from Sigma-Aldrich (Saint Louis, MO, USA). Angiotensin II was purchased from Tocris (Bristol, UK). C21 (MW = 475.2) and EMA401 (MW = 507.2) were synthesized by AGV discovery (Clapiers, France), purified by HPLC (>95%), and characterized by 1H NMR (Figures S3 and S4) and mass spectrometry. Anti-rabbit β3-Tubulin (D71G9) XP^®^ (anti-Tuj1) was purchased from Cell Signaling Technologies^®^ (Danvers, MA, USA). Micro-titer plate (384-well), µClear^®^, was purchased from Greiner Bio-One SAS (Les Ulis, France). Purified mycolactone was obtained and quantified as described in a previous study [9], and re-suspended in DMSO at 5 mg/mL and stored at −20 °C as aliquots in amber glass tubes.

### 4.2. Experimental Animals

Six to eight (6–8) week-old C57bl/6 male mice were purchased from Janvier (Le Genest-Saint-Isle, France) or Daehan biolink (Chungbuk, Korea). In accordance with French regulations (Decret 2013-118—Ministerial Order of 1 February 2013, corresponding to the transposition of the European Directive (2010/063 EU)), the animal experiments were carried out in the animal facility at the Institute Pasteur of Lille (Approval C59-350009 from 21 April 2015). The animal experiments followed the protocol authorized by the Minister of Higher Education and Research, after favorable opinion from the Ethics Committee (CEEA Nord-Pas de Calais/INSERM U1019 No. 00579.01 from 23 July 2014). In accordance with Korean regulations, all procedures for animal use were reviewed and approved by Institutional Animal Care and Use Committee at Seoul National University (SNU-151005-3-2 from 6 January 2017). AT_2_R knock-out mice were provided by Ulrich Wenzel (Hamburg) [37].

### 4.3. Dissection and Dissociation of Mouse DRG Neurons

Adult mice were sacrified after isoflurane anesthesia (Aerrane^®^, Baxter SAS, Guyancourt, France), and the spine was removed. A vertebra was opened carefully by cutting along the frontal plane, to separate the dorsal and ventral sides. DRGs were dissected out from both sides of the spinal cord under the Stereomicroscope. The digestion of connective tissues in the DRGs was carried out for 50 min in a collagenase (380 units)/dispase (2 units) mix, followed by a 0.25% trypsin treatment for further digestion. One milliliter (1 mL) to 200 µL pipette tips (large to small) were used to dissociate the DRG neurons into single cells. Large sized debris were stained using a 100 µm cell strain. The remaining small sized debris and cells were centrifuged on a density gradient composed of 30% BSA solution and Ham’s F-12 Nutrient Mix. Cells whose diameter was greater than 16 µm were counted after trypan blue staining, using the TaliTM Image-Based Cytometer (Life Technologies, Carlsbad, CA, USA).

### 4.4. Culture of DRG Neurons in 384-Well Microtiter Plates

On day one, dissociated DRG neurons were seeded, at a density of 4000 cells per well, in a poly-d-lysine coated 384-well microtiter plate (Greiner Bio-One SAS, Les Ulis, France) using 40 µL neurobasal A medium (supplemented with 10% heat-inactivated FBS, 2% B-27, and 1× Penicillin/Streptomycin). On day two, 10 µL plating medium containing ARA-c and FdU was added on the top of the cells, to a final concentration of 10 µM ara-c and 20 µM FdU, respectively. On day three, the medium in the wells was changed to (50 µL) fresh plating medium, and the cells were incubated overnight.

### 4.5. Immunofluorescence

After 3 days of DRG neurons culture, the cells were fixed with 10% neutral buffered formalin solution for 20 min and permeabilized with PBS + 0.5% Triton X100 for 10 min. The cells were then incubated with blocking buffer (DPBS, 1% FBS) for 1 h prior to incubation overnight with primary antibody at 4 °C. The cells were then washed three times with DPBS and incubated with Alexa-Fluor conjugate secondary antibody for 1 h at room temperature. The cells were again washed three times with DPBS and incubated for 10 min with 5 µg/mL DAPI in DPBS. Finally, the cells were washed for the last time with DPBS. Confocal images were acquired with an automated confocal microscopy system (IN Cell Analyzer 6000, GE Healthcare Bio-Sciences AB, Uppsala, Sweden) using a 10× air lens (NA 0.45). Tuj-1 staining was detected using the 642 nm laser with a 706 nm emission filter, and DAPI staining was detected using the 405 nm laser with a 450 nm emission filter. Four fields per well were recorded.

### 4.6. DiSBAC_2_(3) Assay

DRG neurons were stained for 2 h at 37 °C with pre-warmed plating medium containing 25 µM of DiSBAC_2_(3). The cells were then washed three times with the freshly-prepared sterile imaging buffer (150 mM of NaCl_2_, 5 mM of KCl, 10 mM of HEPES, 2 mM of CaCl_2_, 2 mM of MgCl_2_, 5.5 mM of glucose, and 2.9 mM of sucrose in distilled water, pH 7.5). After washing, the cells were incubated in 40 µL of imaging buffer for 30 min at 25 °C, to equilibrate the membrane potentials of sensory neurons. Prior to the inoculations of the compounds, a first set of images was acquired (Time 0) with the automated confocal microscopy system (IN Cell Analyzer 6000, GE Healthcare Bio-Sciences AB, Uppsala, Sweden). Riluzole, KCl, mycolactone, or DMSO as a negative control was then inoculated into the imaging buffer. After 30 min or 1 h of incubation at 25 °C, a second set of images (Time 1) of the same location was acquired with the confocal microscopy system, using the same cells than those of “Time 0”, with the same settings. The images were acquired with an IN Cell Analyzer 6000 (GE Healthcare Bio-Sciences AB, Uppsala, Sweden), using a 20× air lens (NA 0.45), with an adjustable confocal aperture (1.07 AU). The focus height was adjusted to 3 µm, and a 2 × 2 binning was selected. DiSBAC_2_(3) staining was detected using the 561 nm laser with a 600 nm emission filter and a 0.35 ms excitation time for hyperpolarization or a 0.1 ms excitation time for depolarization. Between 4 to 16 fields per well were recorded.

### 4.7. Image Analysis and Data Management

Each image was processed using image analysis software (version 2.3.0, Perkin Elmer, Columbus, Waltham, MA, USA). As described in previous studies [9,38], two sets of images that were obtained using the same acquisition position, before and after the addition of neuroactive compounds or DMSO, were analyzed. Each pixel was assigned a threshold value according to DiSBAC_2_(3) intensities. The threshold of DiSBAC_2_(3) intensity from glial cells area was defined as a background signal. The pixels with intensities lower than the background threshold were filtered to “0”. Otherwise, the pixels with intensities above the threshold were allowed to define regions of interest concerning DRG neurons. The DiSBAC_2_(3) intensities in the selected regions were measured using the analysis software (Columbus, Perkin Elmer) (Figure S1). The membrane potential changes in the cells, under the effect of tested compounds, were determined for each well as the ratio (Equation (1)) of the sum values defining the intensities of the positive DiSBAC_2_(3) before and after the addition of neuroactive molecules or DMSO:

Ratio = (sum intensity of DiSBAC_2_(3) at Time 0)/(sum intensity of DiSBAC_2_(3) at Time 1) (1)

Neurites were detected on the Tuj-1 positive neuronal cell images based on higher intensity values, compared to the surroundings (Figure S5). The first step of an image’s analysis consisted in the segmentation of nuclei in Tuj-1 positive cell populations. Next, the neurite algorithm (CSIRO Neurite Analysis 2 method in the Columbus image analysis software) was used to detect neurites and neurite trees. For each individual cell, the Maximum Neurite Length as well as the length of the longest neurite attached to the Tuj-1 positive nucleus was recorded.

### 4.8. Cytotoxicity Assay

Mycolactone was added at various concentrations to a 384-well microtiter plate containing the cultivated DRG neurons. After incubation with mycolactone for 24 h, cell toxicity was quantified using the Toxilight bioassay sample kit (Lonza, Walkersville, MD, USA). ToxiLight^TM^ 100% lysis reagent was added as a positive control, allowing for the assessment of the full toxicity. The percentage of viability was normalized (100% viability with vehicle (0 µM mycolactone) and 0% viability with the lysis reagent). Mycolactone in DMSO was diluted 500-fold in complete cell medium prior to the assay.

### 4.9. Competition Binding Experiment

HEK293 cells transfected with 20 ng of SNAP-AT_2_R plasmids were seeded at a density of 50,000 cells per well in 96-well white plates. Twenty-four hours (24 h) after transfection, SNAP-AT_2_R was labeled for 1 h at 37 °C with 100 nM BG-Lumi4-Tb diluted in Tag-lite labeling buffer and washed four times. The fluorescent ligand and compounds to be tested were diluted in Tag-lite labeling buffer. A saturation binding experiment was performed on HEK293 cells overexpressing SNAP tagged AT_2_R using increasing concentrations of fluorescent DY647-AngII ligand. Competition binding experiments were performed using natural ligand fluorescently labelled AngII (DY647-AngII). A fixed concentration of fluorescent angiotensin-II ligand was used as determined by saturation binding experiments (3 nM = Kd) in the presence of increasing concentrations of the compounds to be tested (mycolactone, EMA401, and C21). In the plate containing the labeled cells, 50 μL of the compounds to be tested were added prior to the addition of 50 μL of fluorescent angiotensin-II. The plates were incubated for 3 h in the dark at 4 °C before signal detection. HTRF signal detection was performed on a PHERAstar (BMG labtechnologies, Champigny-sur-Marne, France). The signal was collected both at 665 nm and 620 nm. The HTRF ratio was obtained by dividing the acceptor signal at 665 nm by the donor signal at 620 nm and multiplying this value by 1000. The data points were obtained in triplicates, and fitting was performed using GraphPad Prism (version 5.03, GraphPad Software, Inc., San Diego, CA, USA). The Kd and Ki values are represented as average values for three independent experiments.

### 4.10. Electrophysiology

Whole-cell patch-clamp was performed using an EPC-10USB amplifier (HEKA, Lambrecht, Germany) and the PATCHMASTER software (HEKA, version 2 × 90.1). The resistances of the patch electrodes were 2–5 MΩ. Cells harvested on a 12 mm coverslip were current-clamped close to their resting potential (−55 to −65 mV in average). The pipette solution was composed of K-gluconate 145 mM, MgCl_2_ 2 mM, CaCl_2_ 1 mM, EGTA 10 mM, HEPES 5 mM, and K_2_ATP 5 mM, adjusted to pH 7.2–7.3 with KOH; osmolarity was 300 mOsm. The extracellular solution for the current-clamp experiments contained NaCl_2_ 140 mM, KCl 5 mM, MgCl_2_ 1 mM, CaCl_2_ 2 mM, HEPES 10 mM, and glucose 10 mM, adjusted to pH 7.4 with NaOH; osmolarity was 310 mOsm. The membrane potential was continuously monitored for 20–25 mins after Riluzole or KCl application.

### 4.11. Statistics

Statistical significance (*p*-value) was evaluated with a two-tailed unpaired *t*-test, Mann–Whitney test or ANOVA using Dunnett’s Test (GraphPad Software, Inc., San Diego, CA, USA).

## Figures and Tables

**Table 1:** Cytotoxicity induced by mycolactone in different cell types.

Cell Type [Reference]	Cytotoxicity by Mycolactone A/B		
	Concentration	Incubation Time	% of Cytotoxicity
Peripheral blood lymphocyte [18]	1 µg/mL	90 h	40%
Mouse fibroblast (L929) [19]	15 µg	4 h	>80%
Mouse fibroblast (L929) [20]	60 ng/mL	48 h	75%
Mouse fibroblast (L929) [21]	15 ng/mL	48 h	100%
Peripheral blood human neutrophils [19]	5.3 µg	4 h	>80%
	1 µg	24 h	100%
Keratinocyte stem cell (TAC) [22]	100 ng/mL	24 h	>80%
human keratinocyte line (HaCaT) [22]	1 µg/mL	24 h	>30%
Human hepatoma (Huh7) [22]	1 µg/mL	24 h	0%
Embryonic kidney (HEK293T) [22]	1 µg/mL	24 h	0%
Human primary adult skin keratinocytes [23]	>30 ng/mL	72 h	80%
Endothelial cells (HUVEC) [24]	7.8 ng/mL	4 days	80%
Hippocampal neurons [9]	40 µg/mL	24 h	40%
Mouse bone marrow-derived dendritic cells [25]	100 ng/mL	48 h	50%

**Table 2:** Ligand binding affinities to AT_2_R.

Ligand	Chemical Structure	Ki to AT_2_R
Mycolactone	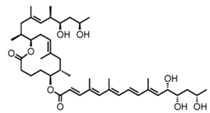	20 µM
AngII	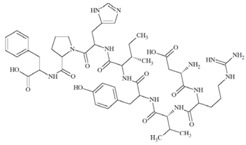	0.1 nM
C21	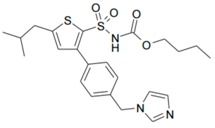	1 nM
EMA401	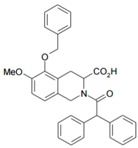	4 nM

## Supplementary Materials

The following are available online at www.mdpi.com/2072-6651/9/7/227/s1, Figure S1: Image analysis workflow for the quantification of positive intracellular DiSBAC_2_(3) signals, Figure S2: Confocal images of DRG neurons from wild-type and AT_2_R-deficient (AT_2_R-KO) mice after loaded with 3.5 µM mycolactone during 1 h, Figure S3: ^1^H NMR spectrum of C21, Figure S4: ^1^H NMR spectrum of EMA401, Figure S5: Image analysis workflow for the measurement of neurite length.
