# Supplementary material for: A Bacterial Toxin with Analgesic Properties: Hyperpolarization of DRG Neurons by Mycolactone
Source: Toxins (Basel). 2017 Jul 18;9(7):227. doi: 10.3390/toxins9070227 (PMC5535174; doi:10.3390/toxins9070227)
Supplement: Supplementary file 1 [file toxins-09-00227-s001.pdf]

Supplementary Materials: A Bacterial Toxin with Analgesic Properties: Hyperpolarization of DRG Neurons by Mycolactone

Ok-Ryul Song, Han-Byul Kim, Samuel Jouny , Isabelle Ricard, Alexandre Vandeputte, Nathalie Deboosere, Estelle Marion, Christophe J. Queval, Pierre Lesport, Emmanuel Bourinet, Daniel Henrion, Seog Bae Oh, Guillaume Lebon, Guillaume Sandoz, Edouard Yeramian, Laurent Marsollier and Priscille Brodin

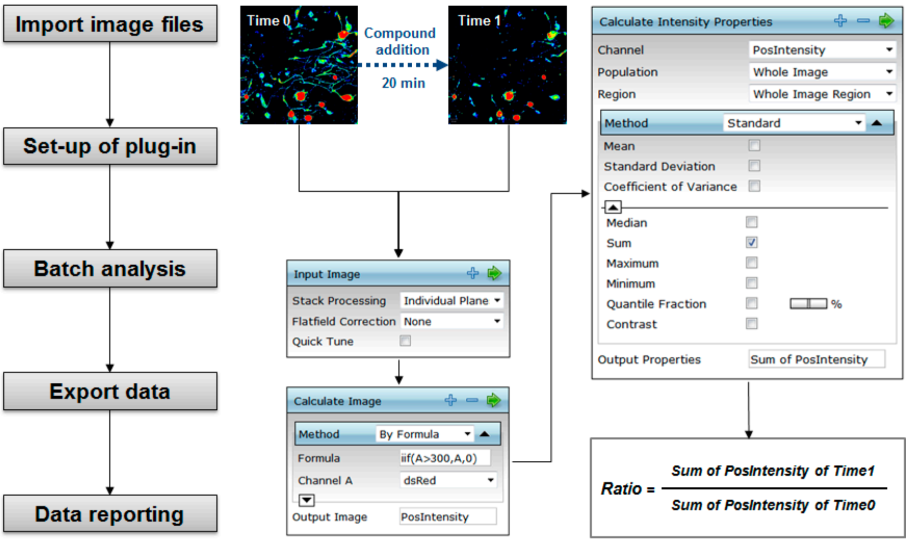

Figure S1. Image analysis workflow for the quantification of positive intracellular DiSBAC2(3) signals.

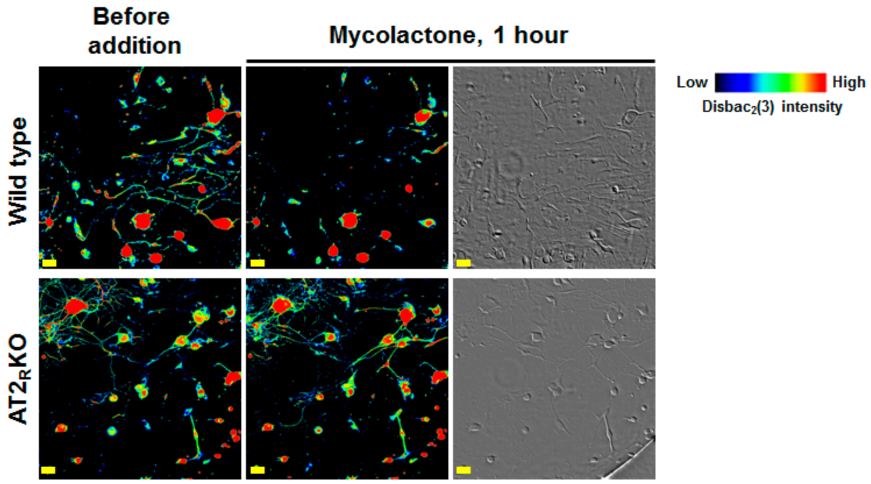

Figure S2. Confocal images of DRG neurons from wild-type and AT2R-deficient (AT2R-KO) mice after loading with 3.5 μM mycolactone during 1 hour; Scale bar = 20 μm.

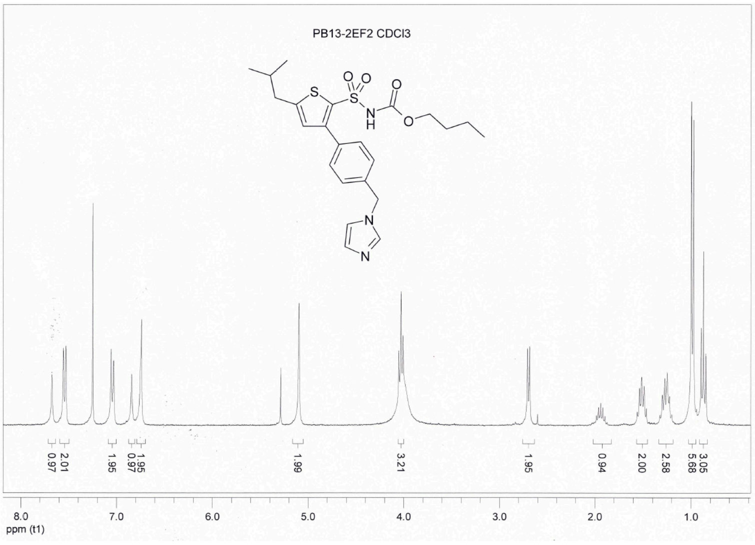

Figure S3. ^{1}H NMR spectrum of C21.

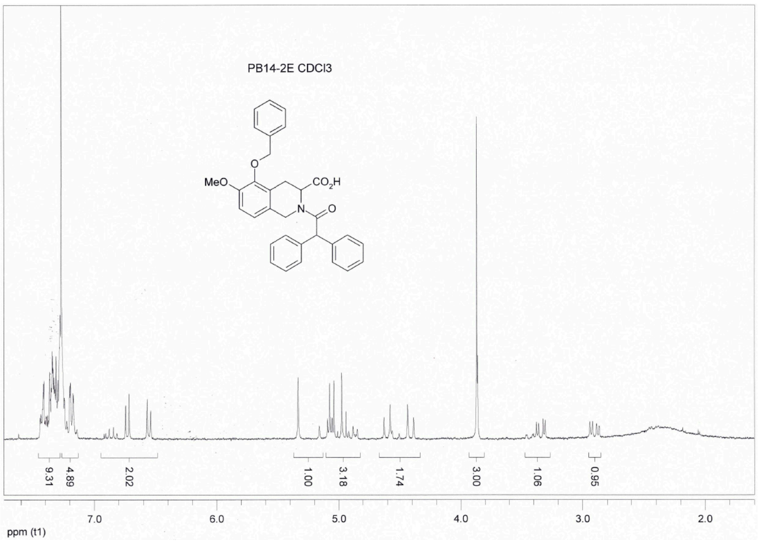

Figure S4. ^{1}H NMR spectrum of EMA401.

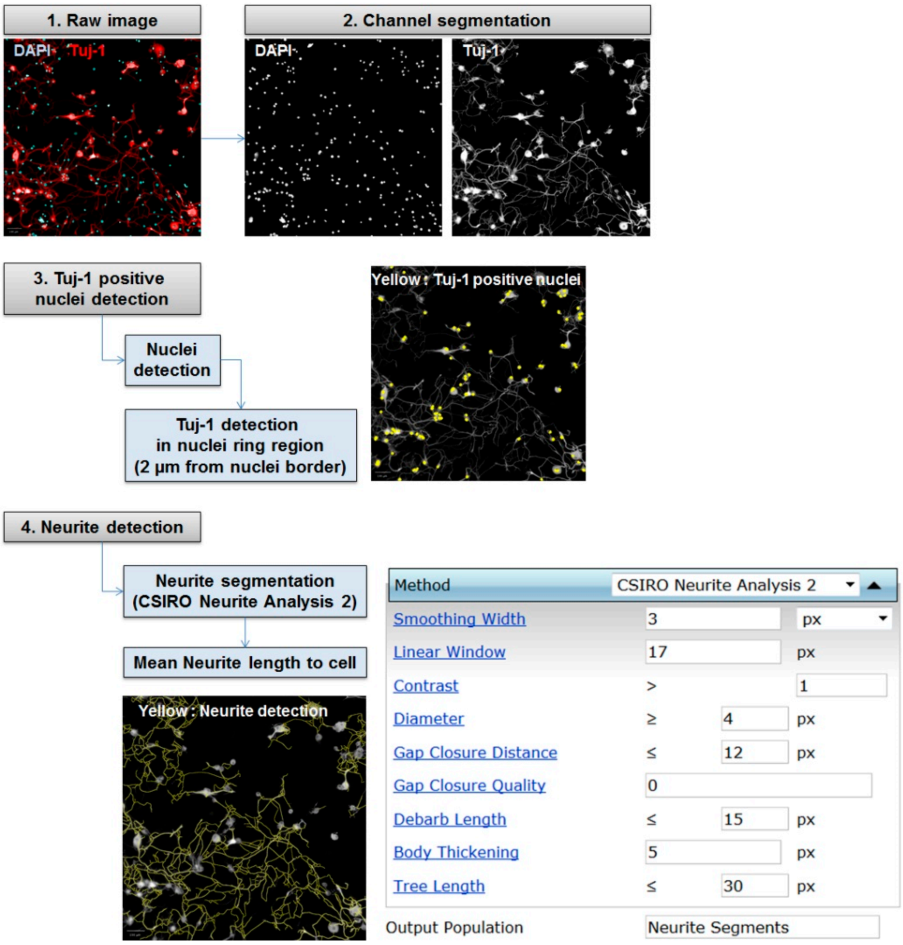

Figure S5. Image analysis workflow for the measurement of neurite length.
